# Vitamin D Modulates Intestinal Microbiota in Inflammatory Bowel Diseases

**DOI:** 10.3390/ijms22010362

**Published:** 2020-12-31

**Authors:** Carolina Battistini, Rafael Ballan, Marcos Edgar Herkenhoff, Susana Marta Isay Saad, Jun Sun

**Affiliations:** 1 Department of Pharmaceutical and Biochemical Technology, School of Pharmaceutical Sciences, University of São Paulo, Av. Lineu Prestes, 580, São Paulo, SP 05508-000, Brazil; carolina.battistini@gmail.com (C.B.); rafael.maluhy@gmail.com (R.B.); marcos.herkenhoff@gmail.com (M.E.H.); 2 Food Research Center, University of São Paulo, Rua do Lago, 250, São Paulo, SP 05508-080, Brazil; 3 Division of Gastroenterology and Hepatology, Department of Medicine, University of Illinois at Chicago, Chicago, IL 60612, USA; 4 Department of Microbiology and Immunology, UIC Cancer Center, University of Illinois at Chicago, Chicago, IL 60612, USA

**Keywords:** antimicrobial peptides (AMP), Crohn’s disease, dysbiosis, epigenetics, inflammation, metabolites, microbiome, micronutrient, nuclear receptor, probiotics, tight junctions, ulcerative colitis, vitamin D, VDR

## Abstract

Inflammatory bowel disease (IBD) is a chronic inflammation of the gastrointestinal tract (GIT), including Crohn’s disease (CD) and ulcerative colitis (UC), which differ in the location and lesion extensions. Both diseases are associated with microbiota dysbiosis, with a reduced population of butyrate-producing species, abnormal inflammatory response, and micronutrient deficiency (e.g., vitamin D hypovitaminosis). Vitamin D (VitD) is involved in immune cell differentiation, gut microbiota modulation, gene transcription, and barrier integrity. Vitamin D receptor (VDR) regulates the biological actions of the active VitD (1α,25-dihydroxyvitamin D3), and is involved in the genetic, environmental, immune, and microbial aspects of IBD. VitD deficiency is correlated with disease activity and its administration targeting a concentration of 30 ng/mL may have the potential to reduce disease activity. Moreover, VDR regulates functions of T cells and Paneth cells and modulates release of antimicrobial peptides in gut microbiota-host interactions. Meanwhile, beneficial microbial metabolites, e.g., butyrate, upregulate the VDR signaling. In this review, we summarize the clinical progress and mechanism studies on VitD/VDR related to gut microbiota modulation in IBD. We also discuss epigenetics in IBD and the probiotic regulation of VDR. Furthermore, we discuss the existing challenges and future directions. There is a lack of well-designed clinical trials exploring the appropriate dose and the influence of gender, age, ethnicity, genetics, microbiome, and metabolic disorders in IBD subtypes. To move forward, we need well-designed therapeutic studies to examine whether enhanced vitamin D will restore functions of VDR and microbiome in inhibiting chronic inflammation.

## 1. Introduction

Inflammatory bowel disease (IBD) is defined as a chronic inflammation of the gastrointestinal tract (GIT) that affects more than six million people worldwide [1,2]. The most common types are Crohn’s disease (CD) and ulcerative colitis (UC) [1]. CD is a segmental, asymmetrical, and transmural inflammation that may affect the whole GIT, but is more frequently observed in the ileum and colon. UC is related to mucosal inflammation from the rectum to the proximal colon [1,3,4]. In fact, IBD has a great impact on the physical, psychological, and social aspects of life, and depression and anxiety are usually increased in these patients. Thus, the management of IBD is of utmost importance for the quality of life of the patients [2].

Several factors are associated with the risk of IBD development, such as country development degree, smoking, sex, age, use of antibiotics or oral contraceptives, lower serum levels of vitamin D, and diet [2,5]. IBD may be triggered by an abnormal immune response to gut commensal bacteria in genetically predisposed individuals and is associated with an impaired intestinal barrier function and a less diverse gut microbiota composition [6,7,8,9].

The gut microbiota is comprised of more than 2000 metagenomic species (MGS) of bacteria distributed throughout the GIT [10]. The population density increases from the stomach to the colon, reaching 10^10^–10^12^ CFU (colony forming units)/mL at the end of the large intestine. Innumerous functions are attributed to the gut microbiota, like metabolism of nutrients from the diet, fiber fermentation, SCFA (short-chain fatty acids) production, vitamin production, barrier function and tight junctions regulation, antimicrobial compounds secretion, immune regulatory, among others [10,11]. Microbial metabolites released by the gut microbiota circulate and may affect the proper function of other organs and systems of the body. Therefore, strategies that address the gut microbiota modulation, improvement of the gut barrier function, and decrease in the intestinal mucosa inflammation are of the greatest significance for IBD treatment [9,12].

Micronutrient deficiencies are often observed in IBD patients, and mostly low levels of vitamin D and zinc, even during disease remission [13]. Observational studies have reported that low levels of vitamin D are directly associated with increased disease activity, mucosal inflammation, clinical relapse, and quality of life. Thus, vitamin D deficiency might be both, the cause, and a consequence of IBD [13,14]. In fact, chronic diarrhea, nutrients malabsorption, low exposure to sunlight, and reduced consumption of vitamin D-fortified foods, like dairy products, are frequent in IBD patients, which may lead to vitamin D deficiency [15].

In this review, we explore vitamin D deficiency and gut dysbiosis in IBD, and the potential use of vitamin D in the management of the disease. In addition, we discuss the epigenetic factors and probiotics involved in IBD and vitamin D/VDR mechanisms.

## 2. Pathogenesis of Inflammatory Bowel Diseases

### 2.1. Genetics

In recent decades, the understanding of the pathophysiology of IBD has markedly evolved. In addition to environmental, genetic, and microbial factors, the pathogenesis of IBD involves the function of cells related to the inflammatory process, such as adipose, epithelial, and endothelial cells, together with regulatory RNAs and inflammasome. For a better elucidation of the disease, a broader approach of all these factors must be performed to clarify the underlying mechanisms that results in the abnormal immune response associated to these diseases [16]. Here, we focus on the main mechanisms related to genetic factors and intestinal microbiota that affect the immune response.

There is an important genetic component that predisposes the development of both UC and CD, and many of these variants are shared in these diseases, thus the mechanistic pathways may be similar. Deficiencies of vitamin D, as well as vitamin D receptor (VDR) polymorphism, could lead to CD susceptibility [17]. A meta-analysis regarding genome-wide association studies (GWAS) showed that, although 110 variants are shared in IBD, there are 23 specific for UC and 30 for CD. The identified loci are enriched for primary immunodeficiencies, reduced circulating T- cell levels, and mycobacterial diseases [18]. In addition, Liu et al. (2015) identified 38 risk loci, increasing the number of known IBD risk loci to 200. These studies may represent a powerful tool for identifying more risk loci for IBD [19].

**Figure 1:**
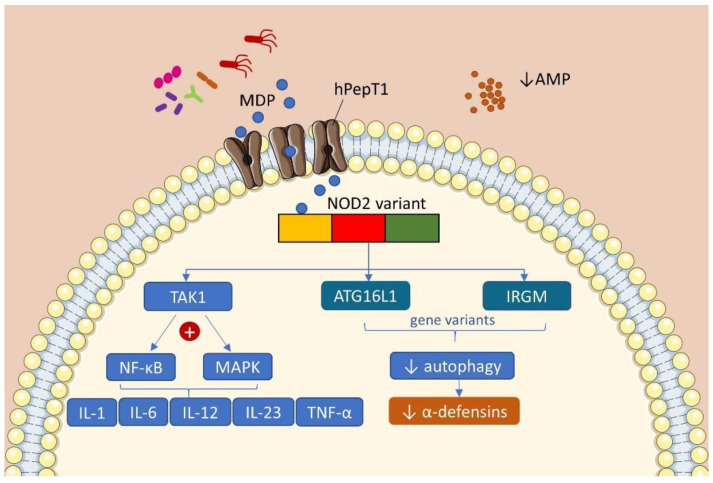
Consequences of the main genetic variants present in the IBD. Bacterial MDP is transported by hPepT1 into the epithelial cell and is recognized by the NOD-2 receptor variant. This interaction activates the kinase TAK1 and its downstream effectors NF-kB and MAPK, leading to the production of pro-inflammatory cytokines. NOD-2 receptor also interacts with genes related to autophagy ATG16L1 and IRGM, reducing the production of AMPs and bacterial killing. In IBD, these mechanisms are impaired due to genetic variants and contribute to the pathogenesis of the disease. (AMP: antimicrobial peptides; ATG16L1: autophagy related 16 like 1; hPepT1: human peptide transporter 1; IRGM: immunity related GTPase M; MAPK: mitogen-activated protein kinase; MDP: muramyl dipeptide; NF-κB: nuclear factor kappa B; NOD2: nucleotide-binding oligomerization domain containing 2; TAK1: transforming growth factor beta activated kinase 1; TNF-α: tumor necrosis factor alpha).

The strongest genetic risk associated with IBD is NOD2 (nucleotide binding oligomerization domain containing 2) [20]. The receptor belongs to the NOD-like receptor (NLR) family and encodes the primary receptor for muramyl dipeptide (MDP) present in all Gram-positive and negative bacteria. NOD2 is expressed in macrophages, Paneth cells, and lamina propria lymphocytes and is pivotal for bacterial recognition. Therefore, it acts in the innate immune response and regulation of commensal microbiota [21]. After binding to MDP, the NOD2 oligomer activates TAK1 (transforming growth factor beta activated kinase 1), which leads to activation of NF-κB (nuclear factor kappa B) and MAPK (mitogen-activated protein kinase), resulting in the production of inflammatory cytokines [22]. Changes in the microbiome with an abnormal NOD2 response can result in an exacerbated immune response and inflammation, which is usually present in CD. Still, NOD2 variants can reduce the transcription of IL (interleukin)-10 anti-inflammatory cytokine [23,24] (Figure 1).

In patients with CD, there is a significant association between NOD2 risk allele count and increased relative abundance of mucosal-associated Enterobacteriaceae, characteristic of dysbiosis in IBD [25]. Additionally, homozygous patients with CD with loss-of-function alleles of FUT2 (Fucosyltransferase 2 gene) presents altered colonic microbiota at both compositional and functional levels, with depletion of *Roseburia* and *Faecalibacterium*, both butyrate-producing bacteria [26].

Other genetic variants associated with autophagy identified by GWAS in CD are ATG16L1 (autophagy related 16 like 1) and IRGM (immunity related GTPase M). Activation of NOD-2 by bacterial MDP in epithelial cells leads to activation of autophagy and increases bacterial killing, a process that is impaired in individuals with CD associated with NOD-2 variants [27]. This further compromises the secretion of antimicrobial peptides, such as α-defensins and other cryptdins [24]. Cryptdins are antimicrobial peptides that are produced by Paneth cells, and their antimicrobial activity is important in reducing infection by pathogenic bacteria such as *Listeria monocytogenes* and Gram-negative bacteria [23,24,28]. Interestingly, both NOD2 [17,29] and ATG16L1 [30] are identified as the downstream target genes regulated by the transcriptional factor VDR. These studies suggest the unique roles of VDR as a genetic factor in IBD.

### 2.2. Microbiota and Immune Response

The human intestinal microbiota include bacteria, virus, fungi, and other microbes. There are approximately 3.8 × 10^13^ bacterial cells and about 100-fold the number of human genes, and the most representative phyla are Firmicutes, Bacteroidetes, Proteobacteria, and Actinobacteria [31,32,33]. Microbiota is shaped before birth and is influenced by the mode of birth, the surrounding environment, breastfeeding, availability of nutrients, and other factors [34,35]. After the cessation of breastfeeding and introduction of food, the infant’s microbiota becomes more similar to that of an adult, and its maturity occurs within 3 years of life [31,36]. Early colonization is essential for the development and maturation of the immune system. Children born by cesarean have delayed colonization and present lower diversity and reduced Th1 (T helper 1) response [37].

Immune receptors, such as Toll like receptor receiver (TLR), NLR, recognize microbe-associated molecular patterns (MAMPs), play a chief role in intestinal homeostasis [38]. The microbial composition is conditioned by the products of the immune and epithelial cells, such as IgA (Immunoglobulin A), mucus, and defensins. Regarding the mucosal immunity, it is regulated by the microbiota. *Bacteroides fragilis* promotes the differentiation of Th1 and Clostridia of T helper Reg (Treg), for example, in a symbiotic relation [39].

The immunological profile of IBD patients is a combination of Th1 and Th1/Th17 in CD and atypical Th2 (T helper 2) in UC [40]. An increase in the pro-inflammatory cytokine IL-17 is observed in the intestinal mucosa and blood, especially in patients with CD. Because Th17 cells produce IL-22 and IL-21, it promotes IFN-γ (interferon gamma) production and Th1 response. In UC, an immune response Th-2 is characterized by the production of IL-5, IL-13, and IFN-γ. There is still disagreement regarding the pattern of cytokines secreted in different diseases and studies showed that the cytokine profile does not always match the type of immune response [41].

In IBD, there is an increased immune response against microbial antigens. This is noted by the circulating levels of antibodies against microbial antigens and glycans. Several studies have pointed out differences in the composition of the microbiota between the IBDs. The patients present an imbalance related to microbial diversity and relative abundance of specific bacteria, namely dysbiosis [42].

Increased intestinal permeability is frequent in CD and UC [43]. A defect in the intestinal barrier could be a primary cause of immunopathogenesis in IBD since increased permeability facilitates the absorption of food and microbial products able to induce an exacerbated immune response and lead to inflammation [44,45]. This is possibly due to a change in the mucus layer in the intestinal lumen. In patients with CD, a reduction in the expression of MUC3, MUC4, and MUC5B mRNA (MUC = mucin; mRNA = messenger RNA) in the ileal mucosa and MUC1 mRNA in the inflamed ileum has already been observed [46,47]. Additionally, the colon mucus of animals that develop UC spontaneously and patients with active UC has been shown to allow bacteria to penetrate and reach the intestinal epithelium [48]. The variants of the NOD2, JAK2 (tyrosine-Protein Kinase JAK2), MUC1, and MUC13 genes are associated with impaired intestinal barrier function and may predispose to infectious and inflammatory diseases and handle an abnormal immune response to luminal antigens [49,50,51].

In comparison to healthy individuals, the microbiome of individuals with IBD fluctuates more over time and show an increase in bacteria of the Proteobacteria phylum, such as Enterobacteriaceae and adherent invasive *Escherichia coli* [52]. Moreover, *Pasturellaceae*, *Veillonellaceae*, *Fusobacterium* species and *Ruminococcus gnavus* are increased, and *Bacteroides*, *Roseburia*, *Suterella*, *Bifidobacterium* species and *Clostridium* groups IV and XIVa are reduced in patients with IBD [53]. Patients with CD usually present a reduction in the phylum Firmicutes, especially *Faecalibacterium prausnitzii*, which is reduced in relative abundance in the stool, and increased abundances of Bacteroidetes and Proteobacteria. In UC, the gut microbiota is characterized by the low abundance of butyrate-producing bacteria, and a high ratio of *B. fragilis*/*F. prausnitzii* is associated with a weaken anti-inflammatory response [42,54,55]. There is evidence that low level of *F. prausnitzii* in stool is associated with relapse in patients with CD in remission. In addition, *F. prausnitzii* induces to a tolerogenic cytokine profile, enhancing IL-10 secretion and reducing IL-12 and IFN-γ [53].

The diversity of viruses and fungi are also reduced in the gut microbiome of IBD patients. A reduction in *Saccharomyces cerevisiae* and an increase in *Candida albicans*, *Candida tropicalis*, *Clavispora lusitaniae*, *Cyberlindnera jadinii*, and *Kluyveromyces marxianus* is commonly observed [53]. Alterations in gut mucosal virome in UC, with increased abundance of Caudovirales bacteriophages and lower diversified virome, correlates with intestinal inflammation and represents a field to be explored to develop new approaches in IBD [56].

## 3. Vitamin D Critical Role in IBD

### 3.1. Mechanisms of Action of Vitamin D

**Figure 2:**
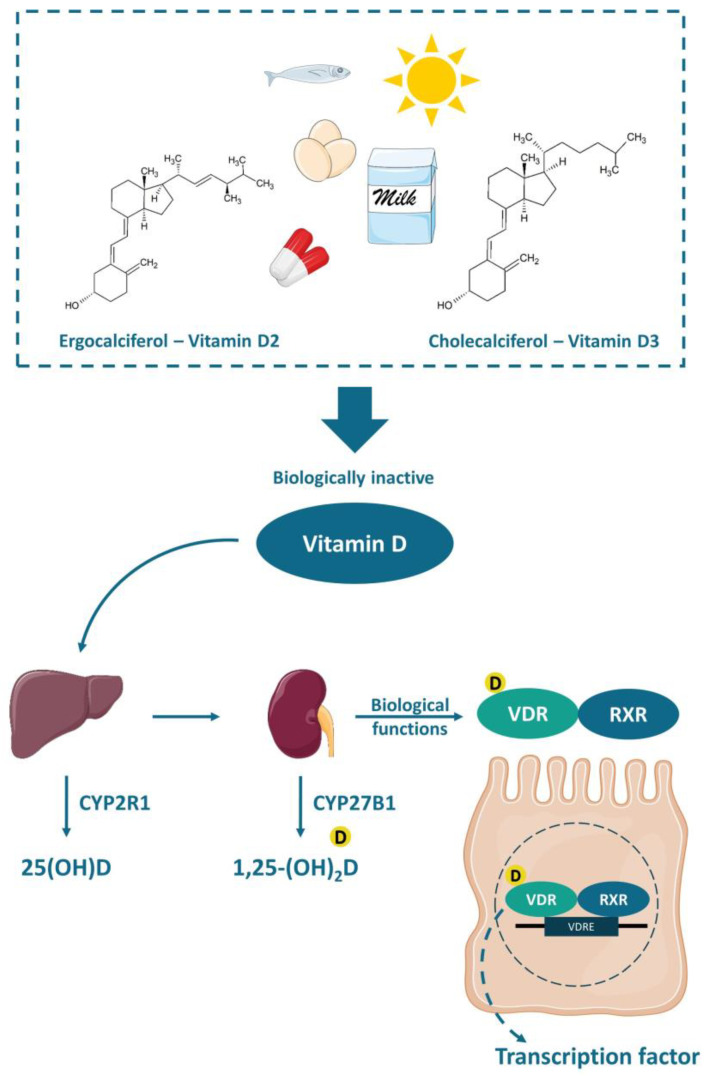
Chemical structure and activation of vitamin D. The vitamin D obtained by the exposure of skin to sunlight or consumed in food or supplements is transported to the liver and converted to 25(OH)D (25-hydroxyvitamin D) by the enzyme 25-hydroxylase (CYP2R1). Thereafter, a second hydroxylation occurs in the kidneys by the enzyme 1-α-hydroxylase (CYP27B1) generating the active vitamin D (1,25-(OH)_2_D or 1,25-dihydroxyvitamin D), which biological functions are mediated by the VDR (vitamin D receptor). The VDR bounded to 1,25-(OH)_2_D forms a heterodimer with the retinoic acid receptor (RXR), which in turn attaches to the vitamin D-response element (VDRE) acting as a nuclear transcription regulator.

Vitamin D is a fat-soluble vitamin that can be found in two different chemical structures: cholecalciferol (vitamin D_3_) or ergocalciferol (vitamin D_2_). It can be obtained either by exposure of the skin to UVB (ultraviolet B) rays from the sun, when the 7-dehydrocholesterol, present in the skin, is converted to cholecalciferol, or by the consumption of some fatty fishes, sun-exposed mushrooms, fortified foods—mostly dairy products, or even by supplements. Vitamin D is transported to the liver by the circulation and transformed into 25(OH)D (25-hydroxyvitamin D), the main circulation form and vitamin D status marker, by the enzyme 25-hydroxylase (CYP2R1). Nonetheless, the 25-hydroxyvitamin D should have another hydroxylation in the kidneys by the enzyme 1-α-hydroxylase (CYP27B1), where it is converted to calcitriol or 1,25-(OH)_2_D (1,25-dihydroxyvitamin D), the active form of the vitamin [57] (Figure 2).

The functions of calcitriol in the body are mediated by the nuclear receptor VDR. VDR is expressed in various tissues (e.g., skin, parathyroid gland, adipocyte, small intestines, and colon). The VDR bounded to 1,25-(OH)_2_D forms a heterodimer with the retinoic acid receptor (RXR), which migrates to the cell nucleus and binds to the vitamin D-response element (VDRE) in the promoter regions of target genes, acting as a nuclear transcription regulator [57,58,59] (Figure 2). The VDRE is found in many genes, explaining the mechanisms associated with vitamin D, like autophagy [30], cell proliferation [60], intestinal barrier function [61,62], gut microbiota modulation [30,63,64], and immune functions [65,66], besides the most well-known mechanism, regarding calcium homeostasis and bone health [58,59,63].

Vitamin D immunomodulatory effects are directly related to antigen presenter cells (e.g., macrophages and dendritic cells) and T-cells functions. It seems that 1,25-(OH)_2_D modulates the T-cell differentiation, shifting from a pro-inflammatory Th1 immune response to an anti-inflammatory Th2 immune response, increasing the secretion of IL-4 while decreasing the secretion of IL-2 and IFN-γ. Moreover, 1,25-(OH)_2_D may inhibit dendritic cell differentiation and IL-12 production while increasing IL-10. Additionally, the lack of 1,25-(OH)_2_D harms regulatory T-cells (Tregs) differentiation and weakens its functions, which may trigger autoimmune diseases [67,68,69].

There is no consensus about the ideal circulating level of vitamin D. According to the Institute of Medicine (IOM), for the majority of the population, a minimum 25(OH)D serum level of 20 ng/mL (50 nmol/L) is considered enough, in case of a minimum sun exposure. Meanwhile, the risk of vitamin D deficiency is considered when the 25(OH)D serum level is below 12 ng/mL (30 nmol/L) [70]. Nevertheless, the Clinical Practice Guideline from the Endocrine Society defined vitamin D deficiency as serum level of 25(OH)D below 20 ng/mL (50 nmol/L) and values between 21–29 ng/mL (525–725 nmol/L) are considered as vitamin D insufficiency [71]. These thresholds of vitamin D serum levels were established for bone health. However, it is known that vitamin D deficiency may also be related to certain types of cancer, cardiovascular diseases and hypertension, type 2 diabetes and metabolic syndrome, autoimmune diseases (e.g., type 1 diabetes, rheumatoid arthritis, IBD, CD, systemic lupus erythematosus, and multiple sclerosis), and infectious diseases (e.g., tuberculosis and upper respiratory infections), autism, depression, and others [57,69,70,71]. A recent review has summarized 130 studies and demonstrated an inverse association between vitamin D and the development of several autoimmune diseases, such as CD, UC, SLE, thyrotoxicosis, type 1 diabetes, iridocyclitis, psoriasis vulgaris, seropositive RA, polymyalgia rheumatic, and MS [72]. These studies support that vitamin D plays an important role on different aspects of the immune system. Furthermore, it is important to point out that the exposure to sunlight is the most effective natural source of vitamin D. However, people usually avoid sunlight exposure or use sunscreen due to skin cancer risk and it is difficult to reach the minimum required through the diet, thus supplementation is often necessary [57,70,71].

### 3.2. Implications of Vitamin D Deficiency in Inflammatory Bowel Diseases

Vitamin D deficiency in IBD patients has widely been discussed in numerous studies. It is common for patients with IBD to self-impose dietary restrictions, which is generally associated with insufficient macro and micronutrients in the diet [73]. One study compared patients with inactive or average CD with healthy controls. An inadequate nutrient intake due to the exclusion of food groups, such as milk, vegetables, and grains in CD group was observed [74]. More than a third of the individuals with IBD had BMI (body mass index) above 25, showing malnutrition accompanied by obesity, which may be due to physical inactivity or treatment with corticosteroids. The main micronutrient deficiencies observed in patients with IBD are zinc, iron, vitamin B12, and vitamin D, contributing to a critical condition and influencing on well-being [75,76].

In the meta-analysis conducted by Gubatan et al., the relationship between low levels of vitamin D and the risks of clinically active disease, mucosal inflammation, clinical relapse, and low quality of life scores among 8316 IBD patients from observational studies was evaluated. Low levels of 25(OH)D were significantly associated with an increase in the clinically active disease [UC (pooled OR 1.47, 95% CI 1.03–2.09, *p* = 0.03, I2 = 0%); CD (pooled OR 1.66, 95% CI 1.36–2.02, *p* < 0.00001, I2 = 0%)] and clinical relapse [UC (pooled OR 1.20, 95% CI 1.01–1.43, *p* = 0.04, I2 = 0%); CD (pooled OR 1.35, 95% CI 1.14–1.59, *p* = 0.0004, I2 = 0%)]. Meanwhile, low vitamin D levels were associated with increased mucosal inflammation and low quality of life scores only in CD patients. In fact, mucosal inflammation may lead to malabsorption of vitamin D in CD, thus low levels of vitamin D could be considered as an inflammation biomarker for CD [14]. Accordingly, MacMaster et al. observed that around 30% of 93 IBD patients in remission presented vitamin D deficiency [13]. Together, the use of standard medications may affect the absorption and use of micronutrients. Sulfasalazine, for example, is a folic acid antagonist, which may lead to anemia when used for a long period. Glucocorticoids decrease the absorption and use of calcium, zinc, and phosphorus and impair vitamin D metabolism [77].

According to an integrative review conducted by Rocha et al., malnutrition is associated to hospitalization of patients affected by the disease. Moreover, nutritional status may influence hospitalization in IBD, although no comparison with adequate nutritional status was evaluated [76]. Low or insufficient levels of vitamin D have already been linked to an increased need for hospitalization and surgery in IBD, when compared to normal serum levels [78,79]. This highlights the importance of maintaining levels considered as adequate for vitamin D, since its anti-inflammatory effect is very well studied, and these patients can benefit their well-being.

Supplementation of vitamin D in IBD patients is challenging due to nutrients malabsorption issues, and higher doses are often necessary to achieve the recommended circulating level (above 20 ng/mL, according to IOM) [70]. Nevertheless, it seemed to be a promising complementary treatment that may improve inflammation markers, such as high-sensitivity C-reactive protein (hs-CRP) and erythrocyte sedimentation rate (ESR), suppressing the Th1 immune response, while reduced clinical disease activity index [14,80,81,82,83,84,85,86].

Despite these challenges, Myint et al. published a guide for clinical practice, as a standard of care, aiming to achieve a concentration of 30 ng/mL of 25(OH)D in patients with IBD, using the following protocol: administration of 50,000 IU/week of ergocalciferol or 2000–4000 IU/day of cholecalciferol (2000 IU/day for 25[OH]D < 30 ng/mL or 4000 IU/day for 25[OH]D < 20 ng/mL), recheck after 8–12 weeks, and if the level of 30 ng/mL is reached, provide maintenance dose between 1000–2000 IU/day of cholecalciferol for the few next months and its discontinuation when the disease is quiescent. The authors explain that although it is not yet known whether vitamin D positively influences disease activity, if it helps, reaching the higher 25(OH)D concentration would have a positive impact, otherwise, reaching moderately high concentrations of 25(OH)D would not be as harmful as vitamin D deficiency would be. It is noteworthy that the authors highlight the methodological limitations and heterogeneity of the observational studies available so far, in addition to the high variability of the strength of association between vitamin D levels and IBD activity, which could be influenced by factors such as genetics, disease subtype or environment, and therefore requires further investigation [15]. However, the status of VDR in IBD patients is not considered in regular treatments. If patients with IBD have genetic variations of VDR or dysfunction of VDR in its biological roles, the supplementation of vitamin D may not work as expected.

### 3.3. Vitamin D and Gut Microbiota Modulation

**Figure 3:**
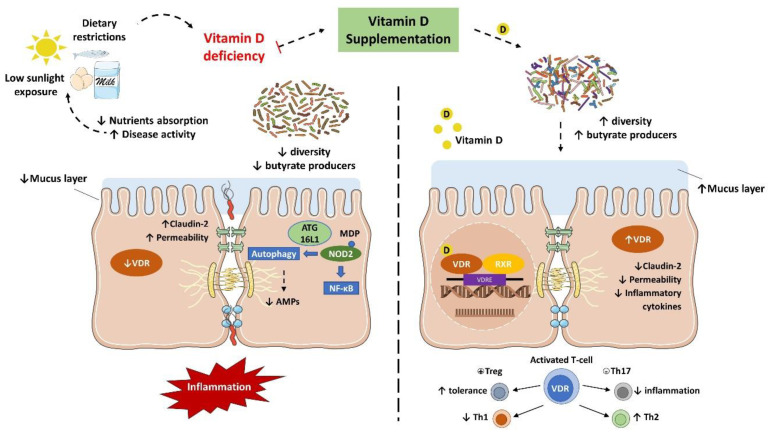
Vitamin D/VDR is involved in the genetic, environmental, immune, and microbial aspects of inflammatory bowel disease (IBD). Thus, the vitamin D supplement and activation of VDR could be considered as a multifunctional factor in IBD treatment.

IBD is characterized by an abnormal immune response to gut commensal bacteria in genetically predisposed individuals, which presents less diverse and imbalanced gut microbiota composition, with less abundance of butyrate producer’s species [7,8,9]. As discussed earlier, vitamin D status is implicated in the severity of IBD while the supplementation seemed to improve the disease status. Nonetheless, it has been suggested that this protective effect is directly related to the gut microbiota (Figure 3). 

Several in vivo studies have shown that vitamin D supplementation and VDR anti-inflammatory effect are directly associated with the gut microbiota. In fact, the downregulation of VDR or the inability to produce the active form of vitamin D were associated with a decrease in *Lactobacillus* in the gut microbiota, while Proteobacteria was increased. Meanwhile, butyrate may improve the VDR signaling, which together with the fact that prokaryotes do not express the VDR highlights that the gut microbiota plays a key role in the host intestinal immune response related to vitamin D mechanisms [30,87,88].

Interestingly, Du et al. reported that in inflammation conditions, the colonic VDR downregulation is associated with an increased expression of local CYP27B1, as a homeostatic protective effect to reduce inflammation and improve VDR signaling. Notably, the gut microbiota plays a critical role in this process. In fact, mice submitted to antibiotic treatment failed to upregulate the CYP27B1 while developed more severe colitis. Meanwhile, LPS treatment stimulated the upregulation of CYP27B1 as well, reinforcing the role of VDR in the barrier function and anti-inflammatory and anti-infection pathways [89].

VDR is known to negatively regulate bacterial-stimulated NF–κB activity [90], and this mechanism may also be an important contributor to intestinal homeostasis and host protection from bacterial invasion and infection. In an experimental colitis model, it was demonstrated that mice with the gut epithelium VDR deletion developed a more severe clinical colitis and worsened epithelial cell apoptosis, leading to an increased intestinal mucosa permeability [61], and promoted the Th1 and Th17 (T helper 17) mucosal response [91]. It suggests that the downregulation of the colonic VDR observed in patients with IBD may be related to impaired barrier functions in the intestine.

The VDR is also implicated in the anti-bacterial functions of Paneth cells. Lu et al. remarkably revealed that the downregulation of VDR and ATG16L1 genes were observed in small intestines tissue from CD patients, as well as a lower percentage and abnormal Paneth cells [92]. Meanwhile, mice with Paneth cells VDR knockout showed a reduction in the relative abundance of beneficial bacteria (e.g., *Lactobacillus*) in the gut microbiota and AMPs release, while were more prone to *Salmonella* infection and DSS-induced colitis [92]. These findings confirm that Paneth cell abnormalities result in a reduced bacterial clearance ability through AMPs, and together with a reduction in autophagy responses could explain the association of dysbiosis and Paneth cell abnormalities observed in individuals with IBD.

Furthermore, higher levels of vitamin D are related to increased serum cathelicidin and reduced inflammation in UC patients. In the meantime, vitamin D may improve the cathelicidin antimicrobial activity against *E. coli* in vitro while showed a protective effect to induced colitis in vivo [93].

There is a lack of human studies evaluating the anti-inflammatory effect of vitamin D associated with its potential in gut microbiota modulation as an adjuvant treatment for IBD management, some of them will be discussed hereafter and are detailed in Table 1. Still, studies with healthy populations have shown promising outcomes about vitamin D and VDR functions in modulating the gut microbiota and improving the immune response [65,66,94,95].

In a pioneering prospective pilot study, Garg et al., 2018 assessed the impacts of vitamin D replacement (40,000 IU, once weekly) over 8 weeks in 25 vitamin D-deficient patients with and without UC in the intestinal microbiota and inflammatory markers. Participants were divided into three groups, active or inactive UC and non-IBD controls. At the end of the intervention, all groups showed an increase in serum concentrations of 25(OH)D, without significant differences. Although no changes in alpha diversity were observed before and after vitamin D replacement in all groups, a subtle reduction in the mucolytic bacteria *Ruminococcus gnavus* was observed. Only participants with UC had an increase in the abundance of Enterobacteriaceae, with no significant change in *E*. *coli* and invasive *Fusobacterium nucleatum*. Despite these findings, the UC group had improved inflammatory markers, such as fecal calprotectin, albumin, and platelet count, together with disease activity. Although a reduction in Enterobacteriaceae was expected, this increase does not imply a worsening in overall profile of microbiota, since this family of bacteria comprises other not harmful and commensal bacteria. These are very interesting results despite the small sample size of the study [80].

Schaffler et al. reported that vitamin D_3_ supplementation (total of 300,000 IU after 4 weeks) altered the gut microbiota composition only in remission CD patients (*n* = 7), and no changes were noted in the healthy controls with vitamin D deficiency (*n* = 10). Throughout 4 weeks, an increase in the abundance of beneficial bacteria like *Alistipes*, *Parabacteroides*, *Roseburia*, and *Faecalibacterium* was observed, even though it was transient. The authors suggested that 4 weeks might have been a too short intervention period to detect a greater change. However, these results suggest that vitamin D administration has potential as an adjuvant therapy for CD patients [96]. It is noteworthy that the reduced abundance of the *Faecalibacterium* genus is commonly associated with both diseases, UC and CD. Its characteristic of producing butyrate has already been shown to be a way to reduce inflammation and promote a balance between Th17 and Treg [98].

In an interesting cohort study, the possible connection between the seasonality of serum vitamin D levels and changes in the microbiome was evaluated. The target population was composed by adults (*n* = 87) with IBD (CD or UC), who lived in regions far from the equator, and both the intestinal mucosa and the fecal samples microbiome were evaluated. After confirming the differences in the concentrations of 25(OH)D, which were higher in periods with higher sun exposure (summer/autumn), some changes in the microbial composition were also observed. In the summer/autumn period, an increase in the abundance of *Pediococcus* spp., *Clostridium* spp., and *Escherichia*/*Shigella* spp. was observed. In contrast, inflammation-related bacterial genera such as *Eggerthella lenta*, *Fusobacterium* spp., *Helicobacter* spp., and *Faecalibacterium prausnitzii* showed lower relative abundance. Unlike other studies, low levels of vitamin D were associated with a more balanced composition of the microbiome. It should be noted that it was not a randomized controlled trial (RTC), but vitamin D levels were correlated with changes in the microbiome in individuals with IBD [97].

There is still limited evidence of vitamin D_3_ in modulating gut microbiota in human IBD. The study designs are heterogeneous together with a substantially small number of patients enrolled in human trials, resulting in inconsistent and controversial outcomes. It is difficult to state an effective dose so far. Meanwhile, it has been suggested that higher doses of vitamin D supplementation may increase the secretion of antimicrobial peptides, contributing to the gut microbiota modulation. Further human clinical trials, with appropriate intervention design, evaluating the impact of vitamin D on the gut microbiota of IBD patients are needed to better understand the mechanisms involved and support the indication of use as a complementary treatment. It is also needed to consider the influence of gender, age, ethnicity, genetics, metabolic disorders (e.g., obesity, diabetes, and NAFLD), and IBD subtype.

## 4. Epigenetics and IBD

Genetics is popularly known as the study of heredity, evaluating the changes in nucleic acids and their performance in organisms. Epigenetics is defined as changes in gene expression or reversible hereditary changes without altering the DNA sequence [99]. A central goal of epigenomics is to understand the gene expression alteration by dietary molecules [100], and it makes a joint focus with nutrigenomics and epigenomics [101]. Epigenetics changes occur in the following ways: DNA methylation, histone modifications, chromatin remodeling, and noncoding RNAs regulation [102].

### 4.1. Epigenetics and IBD

For a long time, a genetic susceptibility in IBD pathogenesis was suggested, and the technological progress in DNA/RNA sequencing allowed many GWAS, and thus, the single nucleotide polymorphisms (SNPs) identification [103,104,105].

The identification of markers for the diagnosis of IBD is of utmost importance, and DNA methylation and miRNAs (microRNA) are special biomarkers for diagnosis at the molecular level. Indeed, studies have shown a strong sensitivity, specificity, and precision of these markers in the diagnosis of IBD [106].

Compared with a healthy control group, patients with IBD showed different changes in the mucosa methylation of the THRAP2, FANCC, GBGT1, DOK2, and TNFSF4 markers. Differences were also observed between patients with CD and UC. CD patients had hypermethylated GBGT1, IGFBP4, and FAM10A4 and hypomethylated IFITM1 when compared to UC patients. Thus, enabling them as markers for differentiating CD and UC [107]. Recently, Kim et al. identified that the fragile histidine triad (FHIT) gene was hypermethylated in patients with CD, therefore a possible biomarker for this disease [108].

### 4.2. Vitamin D/VDR Epigenetic Role in IBD

It is common to associate vitamin D with skeletal homeostasis [109]. However, VDR is linking at hundreds of sites in the genome [110] and is associated with the regulation of more than 60 genes [111,112]. VDR regulates the opening of ion channels, as well as the activity of various enzymes such as kinases, phosphatases, and phospholipases [59].

The role of vitamin D/VDR in the secretion of intestinal mucus may be regulated by the expression of CYP27B1 [113]. In both UC and CD, VDR expression is down-regulated while CYP27B1 is up-regulated [89,114,115]. This reduced VDR expression can also be attributed to the miRNA-346 [114], one of the post-transcriptional mechanisms explained further.

Another way to prevent intestinal inflammation is by regulation of junctional proteins [116]. Although there is a variety of functions and routes that vitamin D/VDR plays a role, there are few studies exploring gene regulation of junctional proteins [117]. Liu et al. showed that VDR binds to histone inhibiting transcription of ZO-1, claudin-5, and occludin genes [118]. Zhang et al. have identified that VDR increases the tight junction protein *claudin-2* as a direct target of the VDR signaling pathway [62]. Interestingly, inflammatory cytokines could also increase the expression of Claudin-2 and enhance intestinal permeability. Thus, lacking intestinal epithelial VDR regulation in inflamed intestine leads to hyperfunction of Claudin-2 and exaggerates the inflammatory responses in intestine [119].

Indeed, epigenetics may explain how environment and genetics might be involved in the development, progression, pathogenicity, and response to treatments. Additionally, epigenetic markers related to immunoregulation, intestinal epithelial barrier, and autophagy are differently expressed among IBD and healthy controls, and between UC and CD patients as well. Moreover, miRNAs may be used as biomarkers for disease assessment in the future, as they are more convenient than endoscopy and biopsies, mainly for patients with active disease [106]. MiRNA are a class of small non-coding RNA (17–22 nucleotides) that regulates gene expression post-transcriptionally. There is a growing interest in understanding and exploring the contribution of miRNAs in common diseases, including IBD [120]. Liu et al. performed a meta-analysis exploring the association of SNPs from miRNAs and IBD. They reported three polymorphisms (rs11614913, rs2910146, and rs3746444) in miRNA-196a2, miRNA-146a, and miRNA-499 in patients with IBD [121].

MiR-196 family showed an overexpression in individuals with Crohn’s disease and downregulates the immunity-related GTPase family M member (IRGM) [122]. IRGM has been shown to be an important factor, and a variant (rs10065172, NM_001145805.1, c.313C > T) has been strongly associated with Crohn’s disease in individuals of European descent [122,123,124,125]. Brest et al. (2011) results provide an explanation for the potential consequence of the *IRGM* c.313C > T polymorphism, and influences predisposition to inflammatory bowel disease in individuals of European ancestry [122]. Additionally, there is evidence for inflammatory-dependent loss of regulation of the autophagy-related protein *IRGM* in individuals with CD, by showing that this synonymous variant alters a miRNA binding site. MiR-30C and miR-130A in T84 cells and in mouse enterocytes showed an up-regulated levels in AIEC infection by activating nuclear factor-κB [126]. Levels of ATG5 and ATG16L1 were reduced by the up-regulation of these miRNAs and inhibited autophagy, and so increasing the inflammatory response [126]. The inhibition of these miRNAs in cultured cells and mouse blocked the AIEC-induced, thus, CD-associated AIEC suppress the autophagy response to replicate within host cells by dysregulating miR-30c and miR-130a expression [126].

In addition, other miRNAs expression profiles changed during IBD. Among them, are the following: miRNA-21, miRNA-122a, miRNA-155 or miRNA-150, which have been associated to intestinal epithelial permeability [127,128,129,130]; and miRNA-126, miRNA-146a or miRNA-155, which are linked to innate and adaptive immune response in intestinal inflammation [131,132,133].

The most studied miRNAs in IBD are miRNA-21, miRNA-155, and miRNA-31 [134,135,136,137,138,139,140]. The association of miRNA-21 and IBD has been the subject of several studies [141]. MiRNA-21 is upregulated in the serum and colonic mucosa in UC and is related to the impairment of tight junctions in intestinal epithelial cells by inducing the degeneration of RhoB mRNA [142]. Moreover, the overexpression of miRNA-31 in tissues of patients with IBD and in mice with colitis induced by dextran sulfate sodium (DSS), reduced IL7R and IL17RA inflammatory cytokine receptors and signaling proteins [143]. Meanwhile, in another study using a mice DSS colitis model, it was demonstrated that miRNA-155 binds directly to SHIP-1 mRNA, responsible for regulating cell membrane traffic [144]. However, these miRNAs act directly at IBD, and none of them related to VDR functions. In fact, James et al. (2020) reported several miRNAs associated with IBD, however, none of them target VDR [141].

Using TargetScan (http://www.targetscan.org), a miRNA database, Agarwal et al. have verified that VDR is regulated by miRNA-23, miRNA-124, miRNA-125, miRNA-302, miRNA-372, miRNA-373, and miRNA-506 [145]. Among these miRNAs, only miRNA-124 was previously associated with IBD, where it has been shown that the reduced levels of this miRNA in colon tissues of children with UC increased the expression of STAT3, as miRNA-124 acts by regulating this protein [146]. Yang et al. (2017) showed that there is a positive regulation of miRNA-23 when cells were treated with high glucose (HG) and miR-23 affected the process of HG-induced epithelial-mesenchymal transition (EMT) in HPMCs by targeting VDR. Although not directly related to IBD, this study is a pioneer in elucidating the role of a miRNA that targets VDR in intestinal epithelial cells, that is, starting from a search on TargetScan and identifying miRNA-23 targets, in this case, VDR, the authors aimed to study the relationship of this miRNA with VDR in the process of intestinal fibrosis [147].

Chen et al. (2017) investigated the role of the lncRNA H19 in the tumorigenesis of many types of colon cancer. They reported that VDR signaling was able to inhibit the expression of H19 through regulating C-Myc/Mad-1 network, and H19, in which turn, was able to inhibit the expression of VDR through miRNA-675. These data showed not only the interaction of a miRNA and VDR but also of another class of ncRNA [148]. To date, there is no study that shows the role of a miRNA by regulating VDR directly in the pathogenesis of IBD, however, miRNA-23 and miRNA-675 seem to be the most promising for studying the role of miRNAs in VDR interaction in IBD.

### 4.3. Probiotics in Regulating Vitamin D/VDR

The potential use of probiotics for microbiota modulation and IBD management have been studied for years [149,150,151]. Ryan et al. reported that inflamed and non-inflamed colonic segments in CD and UC differ in microbiota composition and epigenetic profiles [7]. Moreover, it has been suggested that probiotics may modulate the expression of miRNAs [152].

Importantly, the proper function of VDR is crucial for probiotic anti-inflammatory effects, while probiotic consumption may improve the VDR status as well [153]. Lu et al. reported that the anti-inflammatory and anti-infectious activity of lactobacilli strains isolated from Korean kimchi depends on the VDR expression [154]. Yet, in an earlier study, Lu et al. investigated the tissue-specific role of intestinal epithelial VDR apoptosis and autophagy. The authors concluded that VDR loss impairs autophagy and enhances cell death through apoptosis. They suggested that this mechanism is mediated by the action of vitamin D in ATG16L1 and Beclin-1, which promotes cell survival and thus an anti—inflammatory role in the intestine [155]. Likewise, *Saccharomyces boulardii* revealed to be a promising probiotic specie for the management of IBD, increasing the expression of miRNA-155 and miRNA-223, whereas decreasing the expression miRNA-143 and miRNA-375 [156].

Furthermore, in a study conducted by Chatterjee et al., it became clear that the VDR signaling affects both the microbiome and the metabolomics profile. Indeed, impaired VDR together with a high fat diet (HFD), promoted a significant impact on bile acid metabolism, which was more pronounced in female mice. In addition to microbiome regulation, long chain acylcarnitines (LCACs), tocopherol, and equol metabolisms were also influenced by VDR function and HFD. Thus, it can be concluded that both, diet and VDR status, play a role in metabolic diseases, inflammation, risk of colon cancer, and epigenetic pathways [157].

It is known that everyone responds to probiotic treatment differently and the clinical outcomes are still inconsistent [158,159]. Several studies have been carried out to explore the potential of probiotics in the treatment of IBD, its practical application is not yet recommended, and its effectiveness is unknown. Nevertheless, studies have suggested that probiotics may contribute to the increase in circulating vitamin D, presenting a potential synergetic effect to reduce inflammation [96,160]. Our preliminary data indicate that the probiotic anti-inflammatory effect may be regulated by the VDR, whereas probiotics have the potential to improve the VDR signaling and reduce the inflammatory response [161]. Besides, the most recent AGA Clinical Practice Guidelines on the Role of Probiotics in the Management of Gastrointestinal Disorders recommends, in adults and children with CD and UC, the use of probiotics only in the context of a clinical trial due to lack of evidence so far [162].

In summary, emerging studies have pointed out the role of vitamin D/VDR in regulating proteins that are related to IBD, especially promoting transcription factors, such as miRNAs. There is also evidence that probiotics play a role in these modulations. Our recent study has shown that VDR promotes healthy microbial metabolites and microbiome in a tissue specific and gender specific manner [157]. Thus, more mechanisms and needed for future studies on probiotics for the treatment or prevention of IBD.

## 5. Conclusions and Future Directions

Vitamin D/VDR deficiency could be considered as a multifunctional susceptibility factor and is critical in the development and treatment of IBD. According to the guide to clinical practice, the administration of 50,000 IU/week of ergocalciferol or 2000–4000 IU/day of cholecalciferol to patients with IBD aiming to reach levels of 30 ng/mL that could potentially have a positive impact on the disease activity. Vitamin D administration leads to a shift of the intestinal bacterial composition in CD patients, but not in healthy controls. Meanwhile, beneficial microbial metabolites, such as butyrate, may have the potential to regulate the VDR functions.

So far, vitamin D supplementation contributes to the reduction of inflammation in individuals with IBD and can promote changes in the human microbiota. However, studies reported have several limitations, such as the small sample, the unmatched methodology, or even the lack of definition of what would be the composition of a healthy microbiota. Surely, VDR is a crucial factor for gut microbiota homeostasis, having a great impact on the metabolome profile as well. In addition, its proper functions influence several genes associated with inflammation, barrier function, cancer, autophagy, among others. The downregulation of VDR is related to an upregulation of intestinal CYP27B as a homeostatic anti-inflammatory response, while the VDR function is implicated in regulatory T cells and Paneth cells differentiation, as well as in antimicrobial peptides release, and the gut microbiota seemed to play a critical role in these processes. Therefore, more studies to assess the microbiota and microbial metabolites in IBD are needed.

Finally, a different profile of miRNA is expressed in CD, UC, or healthy control individuals and epigenetics markers revealed to be a highly sensitive, specific, and precise tool for IBD diagnosis, therefore a promising and less invasive alternative when compared with endoscopy and biopsies, which are employed nowadays. Moreover, vitamin D plays a role in IBD regulating transcription factors associated with barrier function and immune responses. The exact mechanisms are not well understood and more studies are needed to explore the therapeutic potential of vitamin D/VDR in the gut microbiota modulation and anti-inflammatory effects in IBD at the metabolic, immunological, and epigenetic levels.

VDR is identified as the first human gene to shape the gut microbiome [63]. However, the variations of the *Vdr* gene in human IBD are still unknown. In the future study, we need considering the status of VDR in the patients of IBD, in addition to the serum 25(OH) vitamin D concentration. We need well-designed therapeutic studies to examine whether enhanced vitamin D will restore functions of VDR and microbiome in inhibiting chronic inflammation, as well as to test the appropriate dose by considering the influence of gender, age, ethnicity, genetics, and metabolic disorders in the IBD subtype.

## Figures and Tables

**Table 1:** Summary of published human studies outcomes evaluating vitamin D3 and its modulation of microbiota in inflammatory bowel disease.

Group	Type of IBD	Treatment/Condition	Duration of Study	Outcome	Ref.
Adults *n* = 25	UC active or in remission	Oral pills: Vitamin D_3_: 40,000 IU weekly	8 weeks	↑ 25(OH)D ↓ clinical disease activity ↓ fecal calprotectin ↓ inflammation in active UC Trend in reducing mucolytic species in fecal microbiota	[80]
Adults *n* = 17	CD in clinical remission	Oral: Vitamin D_3_: Day 1—3: 20,000 IU Day 4—28 (alternated): 20,000 IU	4 weeks	↑ 25(OH)D ↑ week 1: *Alistipes*, *Barnesiella*, *Roseburia*, *Anaerotruncus*, *Subdoligranulum* ↑ week 2: *Faecalibacterium*, *Veillonella*, *Blautia*, *Fusicatenibacter*, *Intestinibacter* ↑ week 4: *Lactobacillus*, *Megasphera* ↓ reduced diversity	[96]
Adults *n* = 87	CD and UC active or in remission	Comparison between Seasonal 25-(OH)D circulating levels (supplemented or not)	Summer/autumm (HE) vs. winter/spring (LE)	25(OH)D levels were correlated with changes in microbiome ↓ 25(OH)D → balanced microbiome composition	[97]

CD = Crohn’s Disease; HE = high sunlight exposure; IU = international units; LE = low sunlight exposure; UC = ulcerative colitis.
